# Total Water Intake and Periodontitis in Korean Adults: Evidence from a Nationally Representative Survey

**DOI:** 10.3290/j.ohpd.c_2784

**Published:** 2026-08-26

**Authors:** Hyo-Jin Lee, Hyun-Jae Cho

**Affiliations:** a Hyo-Jin Lee Professor, Department of Dental Hygiene, College of Dentistry, Kangwon National University, Gangneung, Republic of Korea; Research Institute of Oral Science, Kangwon National University, Gangneung, Republic of Korea. Conceptualization, Methodology, formal analysis, wrote the original draft, reviewed and edited the manuscript, approved final version.; b Hyun-Jae Cho Professor, Department of Preventive Dentistry and Public Oral Health, School of Dentistry, Seoul National University, Seoul, Republic of Korea; Dental Research Institute, School of Dentistry, Seoul National University, Seoul, Republic of Korea. Conceptualization, Methodology, wrote the original draft, reviewed and edited the manuscript, approved final version.

**Keywords:** hydration, periodontal disease, periodontitis, sodium intake, water intake.

## Abstract

**Purpose:**

This study investigated the association between total water intake (TWI) and periodontitis among Korean adults and examined whether this relationship differed according to dietary sodium intake.

**Materials and Methods:**

Nationally representative data from adults aged ≥19 years (n = 9547) who participated in the 7th Korea National Health and Nutrition Examination Survey (KNHANES 2016–2018) were analyzed. Total water intake, representing overall hydration exposure from foods, beverages, and drinking water, was estimated using a 24-h dietary recall. Periodontitis was defined as a Community Periodontal Index score ≥3. Dietary sodium intake was categorized as ≤2000 or >2000 mg/day according to World Health Organization recommendations. Multivariable logistic regression analyses accounting for the complex survey design were performed, with sequential adjustment for sociodemographic factors, health behaviors, and systemic conditions. Stratified analyses were conducted by sodium intake level.

**Results:**

Lower total water intake was consistently associated with higher odds of periodontitis. After full adjustment, individuals with the lowest TWI (≤500 ml/day) had significantly greater odds of periodontitis compared with those with higher intake (≥1500 ml/day; odds ratio [OR] 1.42, 95% confidence interval [CI] 1.15–1.76). This association was evident primarily among participants with high dietary sodium intake (>2000 mg/day; OR 1.42, 95% CI 1.09–1.84), whereas no statistically significant association was observed in the low-sodium group.

**Conclusion:**

Lower total water intake is associated with increased odds of periodontitis in Korean adults, particularly among individuals consuming high levels of dietary sodium. These findings are hypothesis-generating and suggest that promoting adequate daily water intake warrants further investigation as a potential modifiable strategy for periodontal health, particularly in populations with high sodium consumption.

Periodontitis is a chronic inflammatory disease that affects the supporting tissues of teeth, resulting in progressive destruction of the periodontium, including the gingiva, alveolar bone, and periodontal ligaments, ultimately leading to tooth loss. It is caused by a complex interaction between pathogenic bacteria and the host immune response and is influenced by several risk factors, including uncontrolled biofilm, smoking, diabetes, and socioeconomic status.^2,3^ Periodontitis is highly prevalent worldwide. In Korea, it was prevalent in approximately 27.4% of the population in 2012-2015.^27^ Periodontitis causes inflammation throughout the body and is thus associated with several systemic diseases including dementia, cardiovascular disease, stroke, and cancer.^5,6,39^

Water and sodium are two critical components in maintaining overall health, and they may also play significant roles in periodontal health.^7,19^ Water intake is essential for maintaining systemic health and is crucial in various physiological processes. It supports several physiological processes, including hydration, digestion, temperature regulation, toxin elimination, joint lubrication, blood pressure regulation, cognitive function, and skin health.^12,21,25^ Adequate hydration supports mucosal integrity, saliva production, and host defense, which are relevant to periodontal health.^18^ Specifically, adequate salivary flow – which depends on sufficient water intake – is essential for oral clearance, antimicrobial activity, and buffering of bacterial acids in the periodontal environment. It has also been proposed that insufficient water intake may contribute to low-grade systemic inflammation, potentially impairing mucosal immune defense and increasing susceptibility to periodontal tissue breakdown, although direct clinical evidence remains limited. Furthermore, high dietary sodium intake may compound the effect of low water intake on oral health through its osmotic effects and potential pro-inflammatory properties, providing a biological rationale for the observed interaction between water and sodium intake in relation to periodontal health. Sodium is a key electrolyte crucial in fluid balance, nerve transmission, and muscle function. Conversely, high dietary sodium has been implicated in systemic inflammation and may adversely affect oral tissues.^23^ Recent studies have suggested that high sodium intake may also be linked to increased inflammation in the body,^10,34^ which could potentially exacerbate periodontal conditions.

Several studies have investigated the relationship between water intake and obesity, and the findings suggested that higher water intake was associated with lower body weight, body mass index, and waist circumference.^13,28^ Adequate water intake has also been linked to a reduced risk of several systemic diseases, including cardiovascular disease, diabetes, and kidney stones.^31,33^ Conversely, high sodium intake was associated with an increased risk of hypertension and cardiovascular diseases.^10,23,34^ The interplay between water and sodium intake may have significant implications for both systemic and oral health. However, evidence directly linking water intake and periodontitis is limited, and the potential effect‑modification by sodium remains unclear. Therefore, this study aimed to determine whether there was a statistically significant association between water intake and periodontitis, considering the influence of sodium intake, using data from the Korea National Health and Nutrition Examination Survey (KNHANES) VII.

## MATERIALS AND METHODS

### Data Source and Study Population

The study data included a subset of the KNHANES conducted by the Korea Centers for Disease Control and Prevention (KCDC) from 2016 to 2018 and data acquired from the 7th KNHANES. The KNHANES has been approved by the Institutional Review Board of the KCDC (2018-01-03-P-A). Written informed consent prior to participation was obtained from all participants.

The KNHANES sampling protocol comprises a complex, stratified, multistage probability cluster survey of a representative sample of the non-institutionalized civilian population in Korea. The survey uses stratified multistage probability sampling units based on geographic area, gender, and age, which are determined using the most recent 5-year national census in Korea. For the 7th KNHANES, 192 primary sampling units, identified using 2010 census data, were selected across Korea. The final sample set for the 7th KNHANES included 4416 households. A total of 24,269 participants aged 19 years provided data on at least one variable from 2016 to 2018. We included adults aged ≥19 years with valid CPI examinations and complete 24‑h dietary recall data (n = 11,026). Finally, 9547 participants with 20 or more teeth were included in the current analyses.

### Periodontal Status

The Community Periodontal Index (CPI) was used to assess the periodontal status, with periodontitis defined as a CPI score of ≥ 3. Briefly, a CPI score of 3 indicates that at least one index tooth site has a probing pocket depth (PD) > 3.5 mm, and a CPI score of 4 indicates a PD of ≥ 5.5 mm. The index teeth were 11, 16, 17, 26, 27, 31, 36, 37, 46, and 47. A specially designed lightweight CPI probe was used following the World Health Organization (WHO) guidelines.^35^ The probe tip was gently inserted at each site, and the total extent of the sulcus or pocket was explored. Four dentists assessed the periodontal status in the 7th KNHANES.

### Water and Sodium Intake

Total water intake (TWI) was calculated from the 24‑h recall as the sum of water from all foods and beverages and derived directly from the KNHANES nutrient database, which provides gram-level estimates of water content from all reported foods and beverages based on standardized food composition tables. Similarly, sodium intake was calculated as the total sodium intake (mg) of all foods consumed during the day from the 24‑h recall and analyzed continuously and as ≤2000 vs >2000 mg/day.

### Covariates

Covariates comprised sociodemographic variables (age, gender, household income), oral and general health behaviors (use of floss and current smoking), systemic health variables (diabetes and obesity), and sodium intake per day. Household income referred to the monthly average family income. This was adjusted for the number of family members and categorized into four quartiles. Sociodemographic variables, oral and general health behaviors, and systemic health status were examined using a self-administered questionnaire.

### Statistical Analysis

Individual, weighted factors were used, and a complex sampling design of the survey was used to obtain the variance. Multivariable logistic regression analysis was conducted to examine the association between the amount of water consumed per day and periodontitis, adjusted for the effects of covariates in the four logistic models (age, gender, household income, use of floss, current smoking, diabetes, obesity, and sodium intake per day). A subgroup analysis was performed to determine the estimates stratified according to daily sodium intake, a confounding factor. The cutoffs for the subgroups were > 2000 mg/d (high) and ≤ 2000 mg/d (low) following the WHO recommendation. The WHO recommends a daily sodium intake of less than 2000 mg/day for adults.^37^ The participants were also divided into three groups by TWI per day (≤ 500; 500-1500; and ≥ 1500 ml per day) for the subgroup analysis. These thresholds were selected a priori based on the distribution of TWI in the study population and to reflect clinically meaningful distinctions. The ≤500 ml/day threshold identifies individuals with markedly insufficient dietary water intake, a cut-point previously used in large epidemiological surveys to define low water intake groups.^32^ The ≥1500 ml/day group served as the reference category representing individuals with relatively higher dietary water intake in the Korean adult population. All statistical analyses were performed using SPSS version 28.0 software (IBM; Armonk, NY, USA).

## RESULTS

The mean age was 44.55 ± 0.31 years in the periodontitis-free group and 56.95 ± 0.33 years in the periodontitis group. The mean age was 51.03 ± 0.40 years in the participants with sodium intake of ≤ 2000 mg and 46.94 ± 0.32 years in those with sodium intake of > 2000 mg. The percentage of men with periodontitis was higher (35.3%) than that of women (23.6%).

Periodontitis was detected in 32.5% of the participants with the lowest daily TWI and in 27.2% and 25.1% of the participants with a daily TWI of 500-1500 ml and ≥ 1500 ml, respectively. Among participants with a daily TWI of ≤ 500, 500-1500, and ≥ 1500 ml, 44.2%, 77.8%, and 92.2% had a daily sodium intake of > 2000 mg. Table 1 shows the characteristics of the study participants categorized according to their periodontal condition and the recommended daily sodium intake.

**Table 1:** Characteristics of study population based on periodontitis and sodium intake

Variables	No-periodontitis (n = 6762)		Periodontitis (n = 2785)		p-value^b^	Low sodium intake (n = 2428)		High sodium intake (n = 7119)		p-value^b^
	n	% (SE)^a^	n	% (SE)^a^		n	% (SE)^a^	n	% (SE)^a^	
Age (n = 9547)	44.55 ± 0.31^c^		56.95 ± 0.33^c^		< 0.001	51.03 ± 0.40^c^		46.94 ± 0.32^c^		< 0.001
**Gender (n = 9547)**										
Men	2497	64.7 (1.0)	1435	35.3 (1.0)	< 0.001	542	13.5 (0.7)	3390	86.5 (0.7)	< 0.001
Women	4265	76.4 (0.9)	1350	23.6 (0.9)		1886	33.4 (0.8)	3729	66.6 (0.8)	
**Household income** ^d^ **(n=9535)**										
< 25%	807	59.9 (1.9)	585	40.1 (1.9)	< 0.001	510	35.9 (1.6)	882	64.1 (1.6)	< 0.001
25-50%	1570	67.7 (1.3)	765	32.3 (1.3)		655	29.3 (1.2)	1680	70.7 (1.2)	
50-75%	2063	74.3 (1.1)	743	25.7 (1.1)		618	22.6 (1.0)	2188	77.4 (1.0)	
> 75%	2316	77.9 (1.1)	686	22.1 (1.1)		641	21.4 (0.9)	2361	78.6 (0.9)	
**Use of floss (n = 9487)**										
No	4606	67.6 (0.9)	2302	32.4 (0.9)	< 0.001	1802	26.4 (0.7)	5106	73.6 (0.7)	0.016
Yes	2118	82.6 (1.0)	461	17.4 (1.0)		606	23.5 (1.0)	1973	76.5 (1.0)	
**Smoker (n = 9488)**										
Never / quit	5803	73.7 (0.8)	2134	26.3 (0.8)	< 0.001	2170	27.4 (0.7)	5767	72.6 (0.7)	< 0.001
Current	922	61.3 (1.5)	629	38.7 (1.5)		239	15.7 (1.1)	1312	84.3 (1.1)	
**Diabetes (n = 9096)**										
Normal	4730	77.7 (0.8)	1375	22.3 (0.8)	< 0.001	1546	25.4 (0.8)	4559	74.6 (0.8)	0.057
Impaired fasting glucose	1276	63.6 (1.3)	800	36.4 (1.3)		485	24.1 (1.3)	1591	75.9 (1.3)	
Diabetes	448	49.5 (2.0)	467	50.5 (2.0)		258	29.4 (1.8)	657	70.6 (1.8)	
**Obesity (n = 9534)**										
Underweight	318	85.1 (2.0)	63	14.9 (2.0)	< 0.001	105	28.6 (2.6)	276	71.4 (2.6)	0.145
Normal	4346	74.0 (0.8)	1564	26.0 (0.8)		1542	26.1 (0.8)	4368	73.9 (0.8)	
Obese	2091	65.9 (1.2)	1152	34.1 (1.2)		778	24.4 (1.0)	2465	75.6 (1.0)	
**Sodium intake per day (n = 9547)**										
Low (≤ 2000 mg)	1692	71.7 (1.2)	736	28.3 (1.2)	0.605					
High (> 2000 mg)	5070	72.4 (0.9)	2049	27.6 (0.9)						
**Periodontitis (n = 9547)**										
No						1692	25.5 (0.7)	5070	74.5 (0.7)	0.605
Yes						736	26.1 (1.1)	2049	73.9 (1.1)	
**Water intake per day (n = 9547)**										
≤ 500 ml	1127	67.5 (1.4)	583	32.5 (1.4)	< 0.001	955	55.8 (1.4)	755	44.2 (1.4)	< 0.001
500-1500 ml	4434	72.8 (0.9)	1766	27.2 (0.9)		1349	22.2 (0.7)	4851	77.8 (0.7)	
≥ 1500 ml	1201	74.9 (1.3)	436	25.1 (1.3)		124	7.8 (0.8)	1513	92.2 (0.8)	
^a^Weighted percentage ± standard error; ^b^obtained using the complex chi-squared test; ^c^weighted mean and standard error; ^d^household income: monthly average family equivalent income (=monthly average household income/√(the number of household members)										

The results of the multivariable logistic analyses of the total group are presented in Table 2. In the fully adjusted multivariable model, participants with a daily TWI of ≤ 500 ml were 1.42 (95% confidence interval [CI]: 1.15-1.76) times more likely to have periodontitis than participants with a daily TWI of ≥ 1500 ml.

**Table 2:** Multivariable logistic analyses of the relationship between daily water intake and periodontitis in the total group (n=9547)

Groups	Model 1		Model 2		Model 3		Model 4	
	Adjusted OR	95% CI	Adjusted OR	95% CI	Adjusted OR	95% CI	Adjusted OR	95% CI
**Total**								
≤ 500 ml	1.48	1.21-1.81	1.33	1.09-1.64	1.40	1.15-1.72	1.42	1.15-1.76
500-1500 ml	1.17	0.99-1.37	1.08	0.92-1.27	1.11	0.95-1.30	1.15	0.97-1.36
≥ 1500 ml	1.00	reference	1.00	reference	1.00	reference	1.00	reference
Data are shown as the adjusted odds ratios (OR) and 95% confidence intervals (CIs). Model 1 was adjusted for sociodemographic variables (age, gender, and household income). Model 2 was adjusted for age and systemic conditions (diabetes and obesity). Model 3 was adjusted for age, health, and oral health behaviors (current smoking, daily sodium intake, and use of floss). Model 4 was adjusted for age, gender, household income, use of floss, current smoking, diabetes, obesity, and daily sodium intake.								

The association between periodontitis and the lowest daily TWI differed according to daily sodium intake (Table 3). Participants with a daily TWI of ≤ 500 ml were 1.42 (95% CI: 1.09-1.84) times more likely to have periodontitis than participants with a daily TWI of ≥ 1500 ml in the multivariable model 4. Daily TWI was not associated with periodontitis in the low-sodium intake group. In the high sodium intake group, the lowest daily TWI was associated with periodontitis in all multivariable models.

**Table 3:** Multivariable logistic analyses of the relationship between total water intake and periodontitis in the daily sodium intake subgroups (n=9547)

Groups	Model 1		Model 2		Model 3		Model 4	
	Adjusted OR	95% CI	Adjusted OR	95% CI	Adjusted OR	95% CI	Adjusted OR	95% CI
**Low (≤2000 mg/day) sodium intake subgroup**								
TWI ≤ 500 ml	1.54	0.89-2.66	1.63	0.93-2.85	1.51	0.88-2.59	1.50	0.85-2.63
TWI 500-1500 ml	1.21	0.71-2.08	1.24	0.71-2.15	1.24	0.73-2.11	1.26	0.73-2.18
TWI ≥ 1500 ml	1.00	reference	1.00	reference	1.00	reference	1.00	reference
**High (> 2000 mg/day) sodium intake subgroup**								
TWI ≤ 500 ml	1.62	1.27-2.07	1.66	1.30-2.12	1.43	1.11-1.83	1.42	1.09-1.84
TWI 500-1500 ml	1.12	0.95-1.32	1.17	0.99-1.40	1.09	0.92-1.29	1.13	0.94-1.35
TWI ≥ 1500 ml	1.00	reference	1.00	reference	1.00	reference	1.00	reference
Data are shown as the adjusted odds ratios (OR) and 95% confidence intervals (CIs). All the models were adjusted for age as a continuous variable. Model 1 was adjusted for sociodemographic variables (age, gender, and household income). Model 2 was adjusted for age and systemic conditions (diabetes and obesity). Model 3 was adjusted for age, health, and oral health behaviors (current smoking and use of floss). Model 4 was adjusted for age, gender, household income, use of floss, current smoking, diabetes, and obesity.								

## DISCUSSION

The current study found lower TWI was associated with higher odds of periodontitis after multivariable adjustment (Model 4 OR for low vs high TWI = [1.42 (95% CI: 1.15-1.76)]). Stratified analyses indicated that this association was evident primarily among participants with high dietary sodium intake (>2000 mg/day; OR 1.42, 95% CI 1.09–1.84), whereas no statistically significant association was observed in the low-sodium group, suggesting that the relationship between estimated water intake and periodontitis may be modified by sodium intake level. From a preventive dentistry perspective, higher estimated daily water intake may contribute to periodontal health by supporting salivary flow, oral clearance, and mucosal defense mechanisms, although the cross-sectional nature of this study precludes causal inference.

Periodontal pathogens and poor oral hygiene can contribute to the occurrence of periodontitis through high inflammatory sensitivity and low immune response in the body in relation to systemic inflammatory conditions such as diabetes and obesity.^4,26^ The risk factors of non-communicable diseases such as tobacco use, unhealthy diet, lack of physical activity, and harmful use of alcohol should be avoided.^38^ Particularly, to prevent and manage chronic diseases, protective behaviors such as avoiding smoking, reducing alcohol consumption, regularly exercising (sufficient physical activity), and having a healthy diet (reduction of salt/sodium intake) should be practiced.

Drinking a sufficient amount of water as a protective behavior may also have a positive effect on health. Hypohydration can affect physical performance, cognitive performance, gastrointestinal function, kidney function, heart function, headache, and the skin.^25^ Dehydration may compromise the immune system by reducing the production of immune cells and antibodies, impairing their ability to defend against pathogens such as viruses and bacteria.^20^ It can also lead to increased production of pro-inflammatory cytokines, which are signaling molecules involved in the inflammatory process. Chronically low water intake may contribute to low-grade inflammation, which is associated with various health conditions such as cardiovascular disease, diabetes, and arthritis.^25^ Sim et al^30^ reported that participants drinking 1.5 liters of water daily could experience beneficial effects on the physiology of the immune system if they drank an average of 300 ml more water. The authors suggested that the approximately 300 ml/day increase in water intake might have beneficial effects, as biological antioxidant capacity was enhanced and oxidative DNA damage was reduced even in the plain-water group.^30^ This indicates that the increased risk of chronic diseases such as periodontitis can be decreased with adequate water intake.

The present findings are broadly consistent with those of Li et al,^19^ who reported an inverse association between plain water intake and periodontitis in a middle-aged and elderly population using NHANES 2009–2014 data. However, an important methodological distinction exists: whereas Li et al examined plain water intake only, the present study used total water intake (TWI) derived from all foods and beverages, consistent with the international standard definition. Despite this difference in exposure definition, both studies converge on an inverse association between water consumption and periodontal disease. The present study extends prior work by additionally examining sodium intake as an effect modifier, finding that the inverse association was statistically significant only among participants with high dietary sodium intake. This interaction has not been previously reported in the context of water intake and periodontitis, and may reflect the compounding influence of high sodium intake on systemic inflammatory pathways,^1,34^ which may amplify the periodontal tissue vulnerability associated with low water intake. In the broader context of periodontal epidemiology, recent data from the 6th German Oral Health Study (DMS 6) confirm that periodontitis remains highly prevalent and progressive in contemporary adult populations, underscoring the importance of identifying modifiable risk factors such as dietary water intake that may complement established preventive strategies.^15,29,40^

Moreover, a state of low water intake is typically associated with increased serum sodium concentration.^25^ Biomarkers of hypohydration, such as high serum sodium and low water intake, can negatively impact health and increase the risk of mortality.^23,24^ Dmitrieva et al^7^ reported that the amount of water in the body and sodium intake have various effects on health. They concluded that increased serum sodium levels due to low water intake might predict a faster rate of biological aging and an increased risk of early development of chronic diseases. The current study therefore conducted a stratified analysis of the association between estimated TWI and periodontitis according to sodium intake level, given that sodium intake may influence physiological water balance and chronic disease risk. The results showed that the association was statistically significantly stronger with higher sodium intake. This indicates that a higher daily sodium intake can more strongly affect the association between water intake and periodontitis than a lower sodium intake. In addition, the results suggest that periodontal inflammatory conditions can be further lowered by increasing water intake. The stronger association observed among individuals with higher sodium intake suggests that estimated TWI may be particularly relevant in populations characterized by excessive sodium consumption. In such populations, insufficient water intake may exacerbate inflammatory responses and negatively influence periodontal health.

The Institute of Medicine concludes that it is impossible to provide estimated average requirements, owing to the extreme variability in water requirements that cannot be explained by variability in different metabolisms, environmental conditions, and activities.^11^ For instance, obese people need more water than non-obese people because water needs vary with metabolic rate, body surface area, and body weight.^22^ Meanwhile, the WHO recommended a daily TWI of 2.2-2.9 l for women and men aged ≥ 19 years.^36^ This means approximately 10 cups of water per day on average. To achieve this recommendation, intake through other fluid sources, in addition to plain water, should be considered. Therefore, fruits and vegetables with high water content should also be consumed. However, most people globally have a lower water intake.^8^ Moreover, Koreans are known to have a high sodium intake. In 2020, the average daily sodium intake of Koreans aged ≥ 19 years was 3413 mg,^17^ more than 1.5 times the WHO recommendation. Therefore, the results of the current study in the Korean population can increase awareness regarding health management and provide objective evidence for reducing the daily recommended sodium intake and increasing water intake in this population.

This study has several limitations. First, cross-sectional data from the KNHANES was used; therefore, causality of the association could not be confirmed. Moreover, reverse causation cannot be excluded, as individuals with periodontitis may reduce fluid intake because of oral discomfort or xerostomia. Second, because the water and sodium intake were determined from 24-h recall data, these factors could not fully reflect the long-term eating habits and the hydration state of the body. Day-to-day variability in dietary intake means that a single 24-h recall may not adequately capture habitual water consumption, potentially leading to exposure misclassification that could bias results toward the null. There are many methods for assessing hydration, but no ideal method has been established because indicators such as median water intake, urine osmolality, serum osmolality, and deuterium dilution differ among them.^9^ Third, the condition of the periodontal tissue was evaluated using the CPI score; this made it difficult to evaluate alveolar bone loss. Additionally, given the high periodontitis prevalence, odds ratios derived from logistic regression should be interpreted cautiously as they may overestimate the true relative risk. Moreover, the heterogeneity of TWI sources (including sugar-sweetened beverages) may have influenced the observed association. Finally, the restriction to participants with 20 or more natural teeth, while necessary for valid CPI assessment, may have introduced selection bias by excluding those with more severe periodontal disease,^14,16^ potentially underestimating the true association. Nevertheless, to the best of our knowledge, this study is the first to evaluate the association between periodontitis and water intake, with sodium intake as a key confounding factor. In addition, efforts were made to reduce biased interpretation of the results by using integrated data for 3 years from 2016 to 2018. These findings generate hypotheses regarding the potential role of adequate daily water intake as a simple, low-cost, and modifiable behavioral factor in periodontal health, and future prospective studies are needed to confirm this relationship, particularly in populations with high dietary sodium intake.

## CONCLUSIONS

Periodontitis was statistically significantly associated with water intake, and the odds of periodontitis were increased in individuals with higher-than-recommended daily sodium intake. Further research is needed to confirm the causality and identify indicators for understanding the mechanism of the association between water intake, sodium intake, and periodontitis.

## ACKNOWLEDGEMENTS

This study was supported by the Cooperative Research Program for Agriculture Science and Technology Development (Project No. PJ017104) from the Rural Development Administration of the Republic of Korea.
